# Supplementary material for: Ocean variability beneath Thwaites Eastern Ice Shelf driven by the Pine Island Bay Gyre strength
Source: Nat Commun. 2022 Dec 21;13:7840. doi: 10.1038/s41467-022-35499-5 (PMC9772408; doi:10.1038/s41467-022-35499-5)
Supplement: Supplementary file 1 — Supplementary Information [file 41467_2022_35499_MOESM1_ESM.docx]

Supplementary information for the manuscript:

**Ocean variability beneath Thwaites Eastern Ice Shelf driven by the Pine Island Bay Gyre strength**

Tiago S. Dotto^1^, Karen J. Heywood^1^, Rob A. Hall^1^, Ted A. Scambos^2^, Yixi Zheng^1^, Yoshihiro Nakayama^3^, Shuntaro Hyogo^4^, Tasha Snow^5^, Anna K. Wåhlin^6^, Christian Wild^7^, Martin Truffer^8^, Atsuhiro Muto^9^, Karen E. Alley^10^, Lars Boehme^11^, Guilherme A. Bortolotto^11^, Scott W. Tyler^12^ and Erin Pettit^7^

^1^Centre for Ocean and Atmospheric Sciences, School of Environmental Sciences, University of East Anglia, Norwich, NR4 7TJ, UK

^2^Earth Science and Observation Center, Cooperative Institute for Research in Environmental Sciences, University of Colorado Boulder, Boulder CO, USA

^3^Institute of Low Temperature Science, Hokkaido University, Sapporo, Japan

^4^Graduate School of Environmental Science, Hokkaido University, Sapporo, Japan

^5^Department of Geophysics, Colorado School of Mines, Golden CO, USA

^6^Department of Marine Sciences, University of Gothenburg, Box 461, 405 30 Gothenburg, Sweden

^7^College of Earth, Ocean, and Atmospheric Sciences, Oregon State University, Weniger Hall 533, 103 SW Memorial Place, Corvallis OR, USA

^8^Geophysical Institute and Department of Physics, University of Alaska Fairbanks, Fairbanks AL, USA

^9^Department of Earth and Environmental Science, Temple University, Philadelphia PA, USA

^10^Centre for Earth Observation Science, University of Manitoba, Winnipeg, MB, Canada

^11^Scottish Oceans Institute, University of St Andrews, St Andrews, KY16 8LB, UK

^12^Department of Geological Sciences and Engineering, University of Nevada, Reno NV, USA

*Correspondence to*: Tiago S. Dotto ([t.segabinazzi-dotto@uea.ac.uk](mailto:t.segabinazzi-dotto@uea.ac.uk))

This file contains:

Supplementary Figures 1-6

**Supplementary Figure 1. Study area and section beneath Thwaites Eastern Ice Shelf (TEIS).** **a,** Bathymetry and thickness of TEIS from BedMachine Antarctica v2, ref.^37^. Cyan stars show the location of AMIGOS3a (blue edge) and AMIGOS3c (red edge), respectively. Dark red line shows the grounding line from BedMachine. Magenta and white lines show the location of section profile shown in panel b. **b,** Section showing the time-mean conservative temperature and time-mean current speed (flow direction follow Fig. 1b) from the AMIGOS sensors relative to the ice and ocean depths. Ice shelf derived from BedMachine is shown in grey and bathymetry in black. White line is the ice shelf draft as observed by ground-penetrating radar in December 2019 (standard processing included stacking variable from 16-32, band pass filtering range approximately 30 to 150 MHz, gain based on mean attenuation of signal, no migration). Cyan stars show the field estimated ice shelf draft from drilling. Note the depth difference of the estimated ice shelf draft by BedMachine (magenta), radar measurement (white), and drilling (cyan stars). Green dot (right) shows the grounding line from BedMachine.

**Supplementary Figure 2. Borehole Conductivity-Temperature-Depth (CTD) profiles.** **a,** Conservative temperature profiles for AMIGOS3a (blue) and AMIGOS3c (red). Time-averaged temperatures for AMIGOS moorings are plotted at their measured depth and the horizontal black bars show the range of values recorded by each sensor. Field estimated ice shelf draft from drilling is shown by magenta dotted lines. The black dashed line depicts the in situ freezing temperature. **b,** Same as panel a, but for absolute salinity. **c,** Same as panel a, but for neutral density. **d,** Same as panel a, but for meltwater content.

**Supplementary Figure 3. a,** Vertical isoline displacements (m) deduced from conservative temperature (blue), absolute salinity (red) and potential density (black) at AMIGOS3c-Upper using the depths of similar values in the borehole Conductivity-Temperature-Depth (CTD) profiles (shown in Supplementary Figure 2) to the mooring (see Methods). Because the temperature profile is not monotonic (Supplementary Figure 2a), early in the time series (January and February) there are two solutions for the isotherm displacement, above and below the mooring depth; we show these two solutions as open circles and triangles, respectively. **b,** Timeseries of absolute salinity (g kg^-1^) from AMIGOS3c-Upper (black) and estimated from the vertical isopycnal displacements (red). **c,** Same as panel b, but for conservative temperature (˚C).

**Supplementary Figure 4. Comparison of water masses recorded by the AMIGOS moorings (black), Conductivity-Temperature-Depth (CTD) stations in Pine Island Bay (PIB; dark blue), CTD stations surrounding the Thwaites Ice Shelf (light blue), and recorded by an autonomous underwater vehicle**^15^ **west (T2, light orange) and north (T3, dark orange) of Thwaites Ice Shelf. a,** Location of the profiles and the moorings. Ice thickness and bathymetry from BedMachine Antarctic v2, ref.^37^. Note that the Pine Island Ice Shelf front is not calved in BedMachine. **b,** Conservative Temperature (Θ)-Absolute Salinity (S_A_) diagrams showing the water masses in PIB, beneath, at the west and north of the Thwaites Eastern Ice Shelf (TEIS). Red lines depict neutral density. Blue dashed rectangle shows the zoom in depicted in panel c. Black solid line is the Gade line used in Wåhlin et al.^15^ and the black dashed line is the in situ freezing temperature. **c,** Zoom in showing the intermediate and deep waters. The deeper AMIGOS sensors record the fresher type of deep water found in PIB, in agreement with previous findings of Wåhlin et al.^15^. **d,** Profiles of S_A_ versus depth, and **e**, profiles of Θ versus depth.

**Supplementary Figure 5. Accumulated conservative temperature (Θ) flux towards the Thwaites Eastern Ice Shelf (TEIS) by neutral density and meltwater. a,** Accumulated Θ flux from AMIGOS3c-Upper by colour-coded by neutral density (kg m^-3^). **b,** Same as panel a, but coloured by meltwater content. The tilt of the black arrows highlights periods of acceleration (upwards) and deceleration (horizontal) of the flow.

**Supplementary Figure 6. Estimated meltwater content error.** Timeseries of meltwater content estimated from AMIGOS. The shading colour shows the error associated with changing the Winter Water end-member (Methods).
