# Supplementary material for: Ocean variability beneath Thwaites Eastern Ice Shelf driven by the Pine Island Bay Gyre strength
Source: Nat Commun. 2022 Dec 21;13:7840. doi: 10.1038/s41467-022-35499-5 (PMC9772408; doi:10.1038/s41467-022-35499-5)
Supplement: Supplementary file 3 — Description of Additional Supplementary Files [file 41467_2022_35499_MOESM3_ESM.pdf]

Description of Additional Supplementary Files

File Name: Supplementary Movie 1

Description: Trajectories of simulated particles released in the Pine Island Ice Shelf (PIIS) cavity (red rectangle) on daily resolution. A total of 200645 particles were released beneath PIIS in the red rectangle in an offline simulation. For illustration purposes, the particles were restricted to meltwater content of 10-25 g kg-1 and depths 250-400 m, based on day 2 of the simulation (when particles leave the cavity), which reduces the total number of particles to 42521. This video is complementary to Fig. 5 of the main manuscript.
