# Supplementary figures and images for: Ocean variability beneath Thwaites Eastern Ice Shelf driven by the Pine Island Bay Gyre strength
Source: Nat Commun. 2022 Dec 21;13:7840. doi: 10.1038/s41467-022-35499-5 (PMC9772408; doi:10.1038/s41467-022-35499-5)

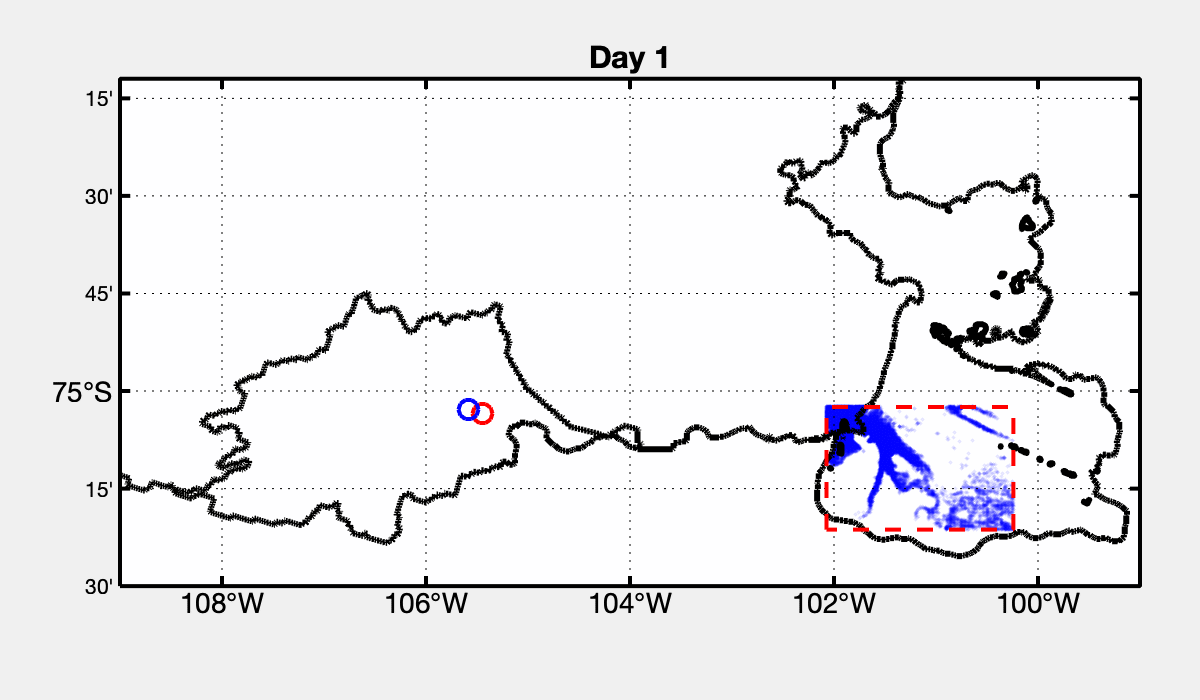

Supplement: Supplementary file 4 — Supplementary Movie 1 [file 41467_2022_35499_MOESM4_ESM.gif]
